# Healthcare professionals’ perceptions and experiences of using a cold cot following the loss of a baby: a qualitative study in maternity and neonatal units in the UK

**DOI:** 10.1186/s12884-020-02865-4

**Published:** 2020-03-18

**Authors:** Paula Smith, Konstantina Vasileiou, Abbie Jordan

**Affiliations:** Department of Psychology, University of Bath, 10 West Building, Bath, BA2 7AY UK

**Keywords:** Cold cot, Cooling technology, Perinatal mortality, Stillbirth, Healthcare professionals, Qualitative interviews, UK

## Abstract

**Background:**

Best practice in perinatal bereavement care suggests offering parents the opportunity to spend time with their baby. Cold cots facilitate this purpose by reducing the deterioration of the body and evidence indicates their wide availability in maternity and neonatal units in the UK. This study aimed to examine healthcare professionals’ perceptions and experiences of using a cold cot following the loss of a baby.

**Methods:**

A qualitative cross-sectional study was designed. In-depth, semi-structured interviews were conducted with 33 maternity and neonatal unit healthcare professionals who worked across three UK hospital settings. Data were analysed using inductive reflexive thematic analysis.

**Results:**

Findings revealed that staff had predominantly positive views about, and experiences of, using a cold cot. The technology was highly valued because it facilitated parents to spend time with their baby and participants reported that it was generally easy to use and smoothly embedded into the clinical environment. Cold cots were deemed useful when mothers were medically unwell and needed time to recover, when parents struggled to say goodbye to their baby, wished to take the baby home, or wanted their baby to stay in the unit instead of going straight to the mortuary. The use of technology was further perceived to be relevant in scenarios of unexpected loss, post-mortem examination and with babies of late gestations or neonates. Despite staff expressing comfort with the delay of visual and olfactory body changes, the coldness of the baby’s body that was accelerated with the use of a cold cot was a major concern as it connoted and possibly exacerbated the reality of death.

**Conclusions:**

Cold cots allow the materialisation of modern bereavement care practices that recognise the importance of continuing bonds with the deceased that is made possible through the creation of memories within an extremely restricted timeframe. Simultaneously, the body coldness concentrates the ambivalence toward an inherently paradoxical death, that of a baby. Training in perinatal bereavement care, including the use of cold cots, would help staff support bereaved parents whilst acknowledging dilemmas and managing contradictions encompassed in death at the time or near the time of birth.

## Background

Although stillbirth rates (i.e., a baby delivered at or after 24 weeks gestational age showing no signs of life, according to the Stillbirth (Definition) Act 1992 [1]) and neonatal mortality rates (i.e., a liveborn baby who died before 28 completed days after birth) have been declining in the UK between 2007 and 2017 [2], in 2018 there were 2958 stillbirths (a rate of 4.00 stillbirths per 1000 total births) and 2028 neonatal deaths (a rate of 2.8 deaths per 1000 live births) across the country [3].

Perinatal death (i.e., stillbirth and neonatal death) has profound short- and long-term psychological effects on parents, such as depression, anxiety, suicidal ideation, guilt, shame and post-traumatic stress [4], with bereaved parents also facing increased likelihood of marital dissolution [5]. Healthcare professionals managing perinatal loss commonly struggle with feelings of guilt, rage, frustration, sense of personal failure, helplessness [6] and the burden of professional responsibility [7] and are at increased risk of developing psychiatric disorders, such as post-traumatic stress disorder, depression, and psychosomatic disorders [6].

To protect parents from traumatisation, parental contact with the stillborn baby was not traditionally permitted in the UK. However, in light of modern theorisations of grief that emphasise the significance of *continuing bonds* with the diseased [8, 9], bereavement care practices started to change during the 1980s towards a direction of supporting parental contact with the dead baby. Current best practice in stillbirth bereavement care suggests that helping parents creating memories, through seeing and holding the baby, taking hand and footprints, items of clothing, locks of hair, photographs and memory boxes, should be offered to parents [10]. Tangible mementos may still be appreciated by parents who might decide not to see and hold the baby and therefore should be collected and stored by the hospital [11] whilst staff should guide parents and sensitively revisit initial decisions of not seeing the baby as the opportunity for parental contact is both fleeting and final [12].

Although bereaved parents have been found to consider the provision of mementos and time spent with the baby as highly important aspects of their care [13], the evidence about the psychological impact of holding the stillborn baby is still sparse and inconclusive [14–16]. Due to the mixed empirical evidence in the area, healthcare professionals are currently advised to offer the opportunity to bereaved parents to spend time with their baby but without forcing it [10]. The technological innovation of cooling facilities – i.e., cold bedrooms and cold cots – has been developed to facilitate parents spending time with their dead child or baby and evidence shows that families’ experience is physically, practically and emotionally beneficial [17, 18].

In the UK, cold cots appear to be widely available in the hospital setting [19, 20] and are used by a significant proportion of bereaved parents who are offered the facility [21]. Despite indications of noteworthy technological diffusion and adoption in the clinical environment and emerging empirical insights on parental experience, very little is known about healthcare professionals’ views and experiences of using a cold cot. To our knowledge, only two studies have examined healthcare professionals’ views of using cooling facilities [22, 23]. The first explored the views of midwives in Sweden who had used a special cot with cooling blocks (which they called ‘Cubitus baby’) with stillborn babies. The analysis of a single, open-ended question included in the questionnaire demonstrated that midwives felt relieved that they were able to give time to parents to say their farewells without being stressed that they must separate the baby from parents [22]. Waught’s [23] interviews with hospice staff in the UK similarly revealed positive attitudes; staff believed that cold facilities were valuable for the bereaved families they cared for and facilitated the process of accepting loss. With a view to building further on this emerging literature, the present study aimed to examine maternity and neonatal healthcare professionals’ perceptions and experiences of offering to bereaved parents and using a cold cot following the loss of a baby.

## Methods

### Study design, research sites and participant recruitment

A qualitative, cross-sectional study was designed. Qualitative research is particularly suitable when the aim is to explore and understand how people experience and make sense of their social realities from their own perspective, providing in this way an account ‘from inside’ [24, 25]. Two Neonatal Intensive Care Units (NICU) and three Central Delivery Suites (CDS), comprising a range of service size and located across three hospitals in the South West of England, were approached by the researchers (PS and AJ) and invited to take part in the present study. All sites had a cold cot available for use. Sites familiar with the use of cold cots and sites less familiar with cold cots at the time of the study were purposively selected to participate.

The study population comprised NICU and CDS staff who have had experience of caring for bereaved parents. Being interested in examining perceptions of the technology as well as experiences of actual use, both staff who have had the chance to use a cold cot and those who have not by the time of the study were eligible for inclusion. A maximum variation sampling in terms of participants’ disciplines (i.e., medical, nursing, midwifery, chaplaincy) was also attempted to represent differing perspectives in bereavement care provision. The study protocol received ethical approval from the Department of Psychology (reference number: 13/140) at the University of Bath and NHS Research and Development (R&D) approval (No: CH/2014/4503) from participating hospital sites.

### Data collection and analysis

Participants were invited to take part in an individual, semi-structured interview which was carried out in line with the designed interview schedule (see Additional file 1). The interview protocol was developed in consultation with health professionals and service users and covered issues concerning usual practice of care after the death of a baby, familiarity with the use of cold cot technology, and staff perceptions of the usefulness and intrusiveness of the technology.

Participants who expressed an interest in the research received an information pack (comprising a participant information sheet and consent form) about the study from the unit manager or researcher and had up to 2 weeks to decide if they wished to participate. Twenty-seven face-to-face and 6 telephone interviews were carried out by PS and AJ, two experienced qualitative researchers, and were audio-recorded with participant consent. On completion of the interview, participants were fully briefed and were offered the opportunity to receive the results of the study which they all accepted. The interviews lasted between 16 and 71 min (average = 37 min) and were carried out during participants’ working time, at their workplace. All participants provided written informed consent before taking part in the interview and had the chance to ask the researcher any questions they might have.

Recorded interviews were transcribed verbatim by University students and researchers checked a random sub-sample of transcripts to ensure quality and precision. Transcripts were subjected to thematic analysis, an analytic technique that is appropriate for the identification of ‘repeated patterns of meaning’ [26]. The analysis proceeded as follows: initially there was a familiarisation process through the repeated reading of transcripts that facilitated immersion into the data. Summaries of each interview transcript were developed, and initial thoughts were noted down at this stage. Then data relevant to our research question were extracted and excerpts of similar meaning were assigned to initial codes. After discussion among the three researchers, a second round of coding took place to refine the definition of codes and ensure thorough and exhaustive assignment of excerpts across the whole corpus of data. Semantically related codes were next grouped together and themes were developed. Initial coding was applied by KV, which was revised and refined in conjunction with PS and AJ. Analytic themes were developed, discussed and finalised among the three researchers.

### Participants

In total, 33 participants were interviewed (the recording of one interview failed leaving 32 usable transcripts). Table 1 provides the details of participant information. All participants were women, and most were either working in CDS as midwives or in the NICU as neonatal nurses, ranging from band 5 to 7. Two participants were doctors, one was the chaplain, and another two were employed as maternity care assistants. Seven participants had used the technology of cold cot whilst 26 had not had the chance to use it by the time of the interviews. One participant had offered the technology to parents, but they did not use it because they spent the time holding and cuddling their baby and then they left.

**Table 1:** Participants’ characteristics (*N* = 33)

Characteristic	***N***
Professions:	
Midwives (i.e. Students = 3; Band^a^ 5 = 1; Band 6 = 5; Band 7 = 7)	16
Maternity care assistants	2
Neonatal nurses (i.e. Band 5 = 4; Band 6 = 5; Band 7 = 3)	12
Doctors (i.e. consultant obstetrician; junior registrar)	2
Chaplain	1
Working at:	
CDS	21
NICU	12
Research Site (RS)	
RS I	9
RS II	13
RS III	11
Gender	
Female	33
Male	0
Had used the cold cot:	
Yes	7
No	26

^a^Bands (ranging from 1 to 9) refer to the pay system that the British National Health Service (NHS) adopts and reflect years of experience, job responsibilities and required qualifications

In the results section below, the participant unique code, the research site from which the participant was recruited and the unit (CDS or NICU) they worked in are provided after each quotation to contextualize the accounts.

## Results

### A. Perceived benefits of using a cold cot

Overwhelmingly, participants expressed positive views about the technology of cold cots, either they were staff who had had the chance to use the technology in practice or not by the time of the research interviews. The delay of body deterioration – that is, the main function of the technology – was considered as the primary functional advantage of using a cold cot. Although participants expressed opposing views as to whether parents do notice their baby’s body changes and to what extent they might get distressed by that, body deterioration in terms of visual (i.e., colour) and olfactory changes was clearly a concern for staff. This was especially true for premature babies who were thought to deteriorate more quickly or when environmental factors (i.e., warmth of rooms in the clinical setting; summer period) accelerated body changes. Having technology available that preserves the body in good condition was consequently considered to be of great benefit.

*I think that the fact that the baby doesn’t change colour so rapidly is very special, I mean I’ve seen parents where they have chosen not to use a cold cot and you can be in a very warm room for about 3 hours and actually the baby can change colour very very rapidly apart from the aroma as well and actually you don’t want that either so a cold cot is brilliant, it really is. (P23, RS III, CDS)*

Staff were clearly able to identify and appreciate the main function of cold cot and some had had the opportunity to observe the outcome of body preservation in practice. Yet, the point at which the technology gained its perceived significance was when staff linked its use to *affording time*. Cold cots were the technological means through which precious time was provided to parents to mourn the loss of their child and have some degree of control over the grieving process. Several participants thought that being able to spend time with the baby was extremely important for parents as it supported them in coming to terms with the situation at their own pace, without the time constraints associated with the physical processes of body deterioration. Cold cots also provided a space for parents to connect with their baby, develop a bond and experience themselves as a family unit with the baby. This was indeed contrasted with common practices of the past when babies were taken away rapidly after delivery and parents did not have the chance to experience feelings of connection, loss and grief. The cold cot connoted a recognition of all these feelings experienced by parents in cases of perinatal mortality whilst the distressing event of the loss was seen to acquire the right level of importance.

*I suppose it helps with the grieving process because departing would be when they were, they would probably never be ready, but when they could break away rather than be broken away to say goodbye. (P04, RS I, CDS)*

### B. Scenarios of technology use

Parents who did want to spend time with their baby was the main precondition for suggesting the use of a cold cot. Participants explained that parents respond differently to the loss of their baby with some wishing to leave the medical environment and return home quite quickly. For these latter cases of parents, the use of a cold cot was not deemed relevant. Discerning parents’ wishes as to whether they would like to spend time with their baby and how much was sometimes challenging for staff and necessitated the adoption of an individualised approach concerning judgments for whom using a cold cot would be beneficial.

*Having the cold cot available for the ladies who are here longer is just, enhances their care I think, you know it’s not for everybody as I say some people almost want to dash out the door a couple of hours after delivery. (P17, RS II, CDS)*

Having judged that the use of a cold cot would be relevant, staff then articulated a series of scenarios in which the deployment of technology was thought to be markedly useful. Such scenarios included situations where the death of the baby was unexpected and when parents had difficulty to separate themselves from the baby and say their goodbyes.

*I think for the mums who can’t quite let the baby go that’s when a cold cot comes in really. (P26, RS III, CDS)*

A further scenario was when mothers were medically unwell and could not hold their baby but still wanted their baby staying with them in the room during mothers’ recovery time. Although participants expressed the opinion that the cold cot could in principle be used with babies of any gestation, as long as parents wished to spend time with them, it was perceived to be even more useful and relevant with babies of later gestations (e.g., term babies) and neonates. The main reason for this was staff’s perception that it is parents of babies nearing term and neonates that are more likely to want to stay with their baby after loss. In cases of older babies, extended family were also more likely to be willing to visit and see the baby. Preserving the baby in good condition, especially when siblings were brought in – often young children themselves – was thus important. Although staff did not exclude the possibility of using a cold cot with babies of earlier gestations, size-related concerns were expressed by two participants, in that the big size of a cold cot compared to that of the baby would not create a sense of ‘nurturing’ environment. As a participant characteristically said*, “the cold cot would have been a huge great big fridge”* (P01, RS I, CDS); for small babies, staff used special sized Moses baskets.

*I think maybe for sort of those babies that are a bit bigger I think it might be useful to use definitely because I think not only do the parents obviously want a cuddle as well but grandparents want to come in don’t they and have cuddles. (P12, RS II, CDS)*

A few participants also suggested that using a cold cot would be useful in cases of post-mortem examination. Although doing a post-mortem examination was not common in the neonatal unit, in cases of unexplained intra-uterine death (IUD) where parents might want to find out the cause of death and also spend time with their baby, the technology was deemed useful. Nevertheless, concerns were occasionally expressed as to whether the use of a cold cot in cases of post-mortem examination would be appropriate in terms of body preservation compared to the routine practice of keeping the baby in the mortuary.

*I guess some of the nurses sometimes just worry that it’s such a hot unit and even though the cold cot is a cold cot I guess you may be worried that will the baby start to decompose slightly or would they be a bit too warm in the outside environment even though they’re lying in a cold cot. (P27, RS III, NICU)*

Two participants expressed the idea that the cold cot could be used with babies in the absence of parents if parents did not want their baby to go to the mortuary for some length of time. The technology was envisioned to replace the function of mortuary, that is, to preserve the body, but the baby would not be physically separated and would remain in the medical unit. Yet, one participant questioned whether having a dead baby placed in a cold cot in a room without the parents being present would be appropriate as it would feel like the baby was abandoned and it might also present challenges for staff who happen to enter that room and encounter a dead body.

Finally, whilst most scenarios of use were envisaged to, and did take place in the medical environment, participants working in the NICU at research site II suggested that the cold cot could be usefully deployed at parents’ home if they wished to take their baby at home before the body goes to the undertakers and instead of keeping it in the mortuary. Despite some staff being supportive and enthusiastic of the idea of cold cot facilitating parents to keep their baby at home, participants also expressed concerns about whether parents would be able to use the technology confidently and effectively without the presence of a healthcare professional. Concerns included: questioning how long the lack of deterioration of the baby’s body would last; the need to have appropriate amounts of sterile water available (applicable to certain models of the technology); and whether support systems (e.g., community teams, helpline) should be first put in place in case of home use so that parents are assisted if they encounter technical or use problems with the technology.

*How do we manage the situation of not a nurse being there but the family being able to confidently use that equipment? (P18, RS II, NICU)*

The work of the staff in the neonatal unit sometimes included an element of palliative care as babies with life-limiting conditions were often being cared for in this environment. One participant mentioned that the cold cot could be used when parents of babies with a life-limiting condition preferred their child to die at home.

The scenario that participants thought that the use of a cold cot was not useful or relevant was when parents wanted to hold and cuddle the baby and did not leave them on a cot during the time they were staying with them.

### C. Introduction of the technology to parents

Several participants expressed the view that identifying the right time at which the technology of cold cot should be introduced to parents was challenging, yet of paramount importance. Interviewees reported that there were no binding guidelines and that the approach they adopted was very much aligned with, and led by, individual parents’ wishes, ways of coping, and pace. For instance, one participant noted that some parents manage the loss of their baby by focusing on the ‘here and now’ whilst others want to know in advance what happens next. In this latter case, participants thought that they were given the opportunity by parents’ questions to introduce the technology. Others thought that when parents expressed the desire to stay with their baby in the medical environment or take them home was the right time to talk about the technology.

*There is never a definite right or wrong thing to say you’re kind of led by the family you’re looking after and you know I think you find your words sometimes as it happens as the situation is in front of you. (P24, RS III, CDS)*

A few participants who had used the cold cot reported that they took the chance to introduce the technology when parents noticed and brought up the issue of body deterioration (i.e., change of colour; stiffness of body). However, one posed the question as to whether the technology should best be suggested when the body starts deteriorating or before that since this participant perceived that the use of a cold cot aimed to prevent deterioration. Others preferred to introduce the technology by focusing on and explaining what it does and how parents are assisted to stay longer with their baby without going into much discussion about the sensitive, and potentially distressing, topic of body deterioration. This facilitated for some a more straightforward introduction that concentrated on the function and purpose of cold cots. Informing parents that the baby’s body will get very cold was for some a necessary and important part of the introduction so that parents anticipate and get prepared for that.

*I think the main important thing to tell parents is the fact that obviously if we do use the cold cot the baby is going to be very cold because I think even if you prepare parents the best you can, that’s my only worry about the cold cot is that the shock to parents of how cold the baby will be and whether that, you know the impact that might have on the parents. (P19, RS II, NICU)*

Finally, one neonatal nurse found that it is appropriate to introduce the technology when she was having discussions with parents about the special room the unit had available for bereaved parents. Although most participants expressed some degree of concern around introducing the technology timely, sensitively and appropriately, one believed that if cold cots were already set up in the clinical setting and had been progressively embedded into the practices of care and routinized – as it is the case with other pieces of medical equipment – its introduction to parents should not pose major challenges and parents would be accepting.

*But could it not just be the norm? I mean I can’t, you know, we put the babies in baskets anyhow so why can’t we put them in cold cots and I, to be honest I don’t think you would be involved in a lot of difficult conversations with parents about because I just don’t actually think the parents are really at, that’s not what’s bothering them at the time, is it? (P08, RS I, CDS)*

### D. Concerns around using and naming the technology

Several participants across the three research sites expressed concern as to whether the use of a cold cot might sharpen and accentuate the reality of death and to what extent it might force parents to face this reality sooner than when they would normally be ready to accept it. According to staff’s view, the coldness and stiffness of the baby’s body that is accelerated with using a cold cot conflicted with instinctual nurturing behaviours which typically aim to keep the baby warm by cuddling it. This tactual sense of coldness and stiffness signalled death and it was questioned whether the use of a cold cot allowed parents to experience transitory time. For some using a cold cot was analogized to ‘putting your baby in the fridge’ or ‘having a mortuary in the room’. Although the usefulness of the technology in terms of delaying body deterioration was clearly recognised, the fact that the technology cools the body of a baby appeared awkward, unnatural and ‘alien’ for both staff and parents. Babies are expected to be warm whilst cold bodies are associated with death. These worries made staff feel that they need to warn parents about the fact that the baby will become *“icy cold”* (P01, RS I, CDS). This was particularly challenging for staff and it was reflected in the uneasiness they expressed about choosing the ‘right’ language to talk about aspects of death that were anticipated to be distressing for parents.

*I think the thing for me was that a baby when it’s born is warm and you cuddle a baby to keep it warm and that’s your nurturing instinct and you don’t want the baby to get cold and what happens with still born babies obviously is they, they get cold because there's nothing but then so your instinct is that you want to keep them warm and what we're doing with cold cots is actually making them cold. And it’s the actual feel, I think it’s true of any dead person, but the feel of a cold baby more so than the look, obviously babies that are dead are blue and pale and don’t look like they are alive, but it’s more the feel of them, the fact that they are icy cold is what really brings home to you that this baby isn't a baby, a normal baby. It’s the stillness and the coldness… (P01, RS I, CDS)*

Participants’ concern around the coldness of the baby was further reflected on the nomenclature of the technology. Several interviewees thought that the term ‘cold cot’ is not attractive and might discourage parents from using the technology as they might get scared at the sound of it. Some even suggested that the name ‘cold cot’ is paradoxical since cots are typically associated with warmth – not with coldness – and are supposed to be an environment that keeps the babies warm. A few participants suggested, and sometimes used, alternative names for the technology such as ‘cooling mattress’, ‘cosy cot’, or ‘special cot’, and preferred to introduce the technology to parents by explaining and talking about how it can help and how it works. Moreover, a few reflected on the possibility of using the term ‘cuddle cot’ that is suggested by the manufacturer of a specific model. Although the name ‘cuddle cot’ was considered more attractive and encouraging, staff were concerned about it being misleading and not representing the reality of the technology. Since participants thought that one of their primary duties is to be honest, yet gentle, with parents and not induce any confusion or misunderstanding, they exhibited considerable creativity in naming the technology in alternative ways that did not sound discouraging but still portrayed the technology.

*We’re calling it a cosy cot. That’s what we’re calling it […]we’re not using the term cold cot because we don’t want it to sound horrible, it’s supposed to be, to help not to make them scared. (P18, RS II, NICU)*

### E. Usability aspects

Despite participants reporting a lack of receiving any formal training on how to set the cold cot up and use it, the majority who had had the chance to use or experiment with the technology found it easy to use and self-explanatory. They also noted that the user instructions it comes with were helpful. Only one participant mentioned difficulty with setting the technology up and explained that she was unsure about whether she was supplying the technology with the right amount of water and why the technology kept beeping whilst trying to set it up.

*We usually get someone in to train us up on, on our equipment so I don’t, I don’t think anybody has come to train us on it…it is a straightforward cot you just kind of plug it in and set it at a certain temperature and there it goes. (P33, RS III, NICU)*

The only problematic aspect that some participants from research sites I and III mentioned was that the technology was quite noisy which seemed to be related to the specific cold cot model available to the unit. Although staff were worried that the noise might be irritating for, and interfere with parents, on occasions the technology had been used the parents did not appear to be annoyed from the noise or pay any special attention to it. Rather, the technology, including its sound, was gradually incorporated into family’s experience of spending time with their baby. Yet, other participants thought that the sound the technology produced when it was being used was very soft and did not create any concerns.

*I mean, the only thing I will say with using it is the ones we have can be, the motor can be quite loud, overnight. (P07, RS I, CDS)*

## Discussion

Cold cots appear to be widely available in maternity and neonatal units in the UK [18, 19] and are largely adopted when bereaved parents are being offered them by healthcare staff [20]. The present study sought to explore maternity and neonatal unit staff’s perceptions and experiences of using a cold cot following the loss of a baby. In line with existing evidence [22, 23], the findings showed that staff had predominantly positive views about, and experiences of, using a cold cot in cases of perinatal loss. The technology through the preservation of the body enabled staff to support bereaved parents spending time with their baby which was considered highly important for the grieving process [23], a view held by both staff who have had the chance to use the cold cot and those who have not by the time of research. Despite a reported lack of training on the equipment, participants generally found the technology easy to use and smoothly embedded into the environment except for the issue of noise which appeared to be model-specific. Further, our findings uniquely identified a range of scenarios of technology use as being articulated by participants themselves. These included cases where the death of the baby was sudden and unanticipated; when mothers were medically unwell and needed time to recover; when parents emotionally struggled to say their farewells, might want to take their baby at home before the funeral, or wanted their baby to stay at the unit for some length of time and not be transferred to the mortuary. Using a cold cot in cases of post-mortem examination was also suggested by some though concerns about the quality of body preservation were simultaneously expressed. The technology was deemed more relevant for babies of later gestations or neonates since it was the parents of these babies who were more likely to want to spend time with their baby. When parents spent the time with their baby cuddling it, offering a cold cot was not considered relevant.

Unlike existing research [22, 23] that conveys an exclusively positive staff attitude toward the technology, the present study identified challenges and concerns alongside the perceived value of the technology. The major concern expressed by staff was that by using a cold cot the reality of death might be sharpened through the coldness and stiffness of the baby’s body. The transitory and highly valued time that the technology might afford to parents to come to terms with their loss was thus challenged and questioned in this instance. The uneasiness regarding the symbolic content around the body coldness that is accelerated through using the technology was further reflected on the nomenclature and introduction of the equipment to parents. Participants strived to come up with terms that balanced the need to convey the reality of the technology without discouraging or scaring parents and adopted an individualised approach as to when and how the technology was most gently introduced. Further, although participants were supportive of the use of cold cots at home by parents, worries were expressed regarding parents’ ability to use the technology effectively and troubleshoot potential technical problems and whether support systems in the community should be first put in place to aid home use. Although in the UK there are no legal reasons to prevent parents from taking their baby home (unless a Medical Certificate of Cause of Death cannot be issued whereby the death must be referred to the coroner) [27], it should be noted that discharging a deceased baby might raise legal issues in other countries. Finally, staff raised concerns in scenarios where the cold cot was envisaged to replace the mortuary. In cases of post-mortem examination participants were unsure about whether the cold cot was functionally equivalent to the mortuary in preserving the body. Staff were finally ambivalent about the appropriateness, for both the dead body and staff, of keeping the baby in the unit within a cold cot whilst parents were absent instead of taking it to the mortuary.

### Strengths and limitations of the present study

To our knowledge, this is the first study that qualitatively examined healthcare staff’s views of, and experiences of using, cold cots in the hospital setting, supplementing relevant research conducted with hospice staff [23]. Using a diverse and large enough sample that allowed thematic saturation, we are confident that the empirical insights derived from the analysis are credible and reflective of a range of views. Our findings regarding the perceived value of the technology resonate strongly with emerging evidence in the area [22, 23] suggesting the transferability of these insights. Large-scale, quantitative research is needed though in relation to the symbolic concerns that our participants articulated about the technology to support their validity and determine their prevalence. Although the present study included questions around aspects of technology usage and related use problems, appropriately designed usability studies that employ observational methods would more sufficiently and closely capture usability issues. Finally, this research only captures healthcare professionals’ views, and specifically those of women; to gain a fuller understanding of the usefulness of, and potential issues around, the technology the bereaved families’ experiences need also to be examined as well as the perspectives of male healthcare professionals managing perinatal loss.

## Conclusions

Despite the apparent diffusion and adoption of cold cots in the clinical setting [19–21], this study identifies little provision of formal training on the equipment leaving healthcare professionals susceptible to use errors. Similarly to other medical technologies, formal training on the use of cold cots should be provided that will minimise potential use errors, increase knowledge and enhance staff confidence in using the technology in a work situation that is emotionally laden [6, 7] and requires delicate management to meet bereaved parents’ complex needs for meaningful care [28].

Arguably, cooling technologies, including cold cots, can be conceived as the technological means through which changing bereavement care practices – shaped by broader shifts in understandings of parental grief [8, 9] – are enabled to materialise. The technology comes to acquire its symbolic value by facilitating the adoption of modern care practices that advise spending time with the baby and creating memories [10]. Nevertheless, the evidence around the impact of seeing and holding the baby on parents’ mental health and wellbeing is still inconclusive [16] with some studies suggesting negative effects [14]. Given the greater exposure to the dead child implied in cold cot use, potential adverse impacts on parents might be exacerbated. Until more conclusive evidence in the area are adduced, caution with offering and using the technology should be exercised. Finally, whilst the purpose of spending time with the baby is well served through the delay of visual and olfactory body deterioration, the concerns narrated about the coldness of the baby’s body possibly condense the distress toward an incomprehensive death, that of a baby whereby the convergence of life and death is fundamentally paradoxical [4]. To acknowledge, make sense of, and better manage these concerns and contradictions, bereavement care training and support for staff is of paramount importance [29].

## Supplementary information

**Additional file 1.** Interview Schedule.

## Data Availability

The datasets generated and/or analysed during the current study are not publicly available due to the sensitive nature of the data, but they are available from the corresponding author on reasonable request.

## Abbreviations

CDS: Central Delivery Suite
NICU: Neonatal Intensive Care Unit
IUD: Intra-Uterine Death
