# Supplementary material for: Healthcare professionals’ perceptions and experiences of using a cold cot following the loss of a baby: a qualitative study in maternity and neonatal units in the UK
Source: BMC Pregnancy Childbirth. 2020 Mar 18;20:175. doi: 10.1186/s12884-020-02865-4 (PMC7079527; doi:10.1186/s12884-020-02865-4)
Supplement: Supplementary file 1 — Additional file 1. Interview Schedule. [file 12884_2020_2865_MOESM1_ESM.docx]

# **Additional File 1**

## Interview Schedule

First of all, I would like to thank you for taking the time to talk to me. As you know we are interested in understanding your experience of caring for parents and relatives who have lost a baby in your unit. I have here some topics which might be useful to discuss, but most important are your experiences.

I am going to record the interview so that I can listen to what you have said at a later date. However, if you want to stop the interview or the tape recorder at any time you can. All the information you provide will be strictly confidential. Your name will not be mentioned in any reports arising from this study.

***Section One – Your experience of supporting families who have lost a baby***

1. So that we can understand your experience can you tell me if you have ever had to support a family who have lost a baby?

*Prompt:* For example, how long ago? Was this something for which you felt sufficiently prepared? If you have not experienced this situation have you thought about how you might manage such a situation?

2. Does your unit have any policies or procedures for managing the loss of a baby?

*Prompt:* Have you had the opportunity to look at them/discuss them with other colleagues? How useful do you think they are/will be? How many bereavements does your unit have per year?

3. Have you had any special training in supporting bereaved parents and families?

*Prompt:* Was this in your original training or post qualification? Is this something that the unit offers or encourages you to undertake?

4. Is there a specialist bereavement health professional that you can access in your unit?

*Prompt:* E.g., bereavement midwife/chaplain/psychologist?

5. If you have a specialist in your unit what do you see their role to be?

*Prompt:* To support parents/family/staff? Long term/short term support? Advice about practical issues such as funeral/finance etc.?

***Section Two – Managing a neonatal death***

In this section we would like to explore your actual or imagined experience of managing the loss of a baby. Please recall or imaging that you are the primary health care professional involved with parents who have just experienced the loss of a baby.

6. Can you talk me through what you would do in this situation?

*Prompt:* Would you undertake any formal tests or actions (to see if the baby was still alive)? Are there any formal procedures to be followed? What would you say to the parents?

7. What would be likely to happen during the first hour after the death of the baby?

*Prompt:* Medical/technical procedures? Care of the mother? Care of the infant? Care of the father?

8. What is likely to happen after this time?

*Prompt:* Suggest holding baby? Taking photos and making memories? Dealing with legal requirements? Contacting other family? Moving to quiet room?

***Section Three – Supporting parents on the unit***

In this section, we would like to consider the longer term management and support of the parents and other family members whilst they are in your unit.

9. Does your unit have a special area/room for parents who have lost a baby?

*Prompt:* Is it away from the normal ward? Do parents have to be moved along public corridors to reach it? Is it self-contained? How long can they use the room? Are visitors allowed/encouraged?

10. Do parents normally spend time with the baby?

*Prompt:* Is this normally just in the room or can they go elsewhere? E.g. hospital garden, mortuary, hospital chapel? Do parents request any special facilities in this time?

11. Do parents ever refuse to spend time with the baby?

*Prompt:* If so, how is this managed?

***Section Four – Use of cold cots***

In this section we would like to discuss the use of cold cots in your unit.

12. Does your unit have cold cots available to use? What make/model is it?

*Prompt:* Have you seen/practiced using the cold cot in your unit? Have you had training in its use? How was the unit acquired (e.g. donated by charity)? How long has the unit had the cold cot? If your unit does not currently have a cold cot, do you have plans to obtain one? How will this purchase be funded?

13. Who are the cold cots primarily used by?

*Prompt:* Are they offered to parents of late/early gestation babies? Who are they introduced by usually? Are they taken home by parents?

14. Have you ever used or how would you introduce the cold cot to parents?

*Prompt:* is it already in the room or does it need to be brought in? Would it be used for all bereaved parents? Do you explain why it is important and how to use it?

15. What are your main impressions of the cold cot in this situation?

*Prompt:* Did you find the cold cot useful? Did the parents/family use it correctly or did they ignore the cold cot? Did you find the cot to be unobtrusive or not? Was it easily set up?

16. If you were to use the cold cot again would you manage the procedure or equipment differently?

*Prompt:* Would you change the location and set up of the equipment? Would you introduce the equipment to parents differently? Would you be more selective which parents you introduced the equipment to?

17. Finally do you have anything to add that would help us to understand your experience of supporting parents and using cold cots in the future?

Thank you for your participation in this study.
